# An Atypical Presentation of Osseous Choristoma in a 63-Year-Old Male: A Lesion Beyond the Common Demographic and Anatomical Norms

**DOI:** 10.7759/cureus.74327

**Published:** 2024-11-23

**Authors:** Chance J Aplanalp, Randall Hansen, Alex L Otto, John H Irlam, Scott McClintick

**Affiliations:** 1 Otolaryngology-Head and Neck Surgery, Kansas City University of Medicine and Biosciences, Joplin, USA; 2 Otolaryngology-Head and Neck Surgery, Freeman Health System, Joplin, USA; 3 Pathology, Freeman Health System, Joplin, USA

**Keywords:** base, choristomas, osseous, root, tongue

## Abstract

Osseous choristomas, characterized by ectopic bone, are rare and typically found in the head and neck, particularly on the tongue. This report describes a unique case of an osseous choristoma in a 63-year-old male with an unusual posterior tongue location. An incidental base of tongue (BOT) mass was discovered during a bronchoscopy for lung biopsy. Physical examination revealed a 3-4 cm pedunculated mass at the root of the tongue posterior to the foramen cecum. The patient had a significant 60-pack-year smoking history. A biopsy confirmed the diagnosis of osseous choristoma. The mass was surgically debulked using an intraoral approach. Histopathology revealed findings consistent with osseous choristoma. Intraoral osseous choristomas are exceedingly rare, with fewer than 100 cases reported. This case is unusual due to the patient's older age, male gender, and the mass's posterior location. Surgical excision remains the treatment of choice, with recurrence being rare. This case adds to the limited literature on osseous choristomas by documenting an atypical presentation. The report underscores the importance of considering choristomas in differential diagnoses, regardless of patient age or lesion location, and highlights the efficacy of patient-centered surgical approaches in managing these rare lesions.

## Introduction

Choristoma describes the presence of histologically normal tissue placed at an atypical location. These are benign lesions that coexist with the native tissue in the correct anatomical location. Choristomas can be characterized by tissue type and anatomical location. There are several case reports of choristomas presenting in the oropharynx, orbit and eye, central nervous system, and middle ear [1-4]. The classification of histologically normal tissue that appears at the abnormal site has an extensive range and presents with no recognizable pattern. For instance, Rinaldo et al. report a choristoma of the middle ear where a salivary gland is recognized [4]. These heterotopic lesions are arbitrary in terms of tissue type and anatomical location.

Osseous choristomas are distinguished by the presence of ectopic bone [5]. Although rare, these bone masses are often found in the head and neck region, specifically on the tongue's surface, the cervical neck region, and the nasopharynx or oropharynx [5-7]. A review on osseous choristomas mentions that females are predominantly affected at a 4:11 ratio and that the average age of presentation is 28.7 years [8]. Adhikari et al. describe that osseous choristomas are often located on the posterior tongue anterior to the foramen cecum near the circumvallate papillae [9]. Here, we present a patient outside the typical osseous choristoma demographic with a mass at an unusual location. Specifically, this patient is a 63-year-old male with an osseous choristoma located at the base of the tongue positioned posterior to the foramen cecum.

## Case presentation

The patient described in this case report is a 63-year-old male who was referred to the otolaryngology department after a base of tongue (BOT) mass was discovered incidentally on a bronchoscopy during a lung biopsy procedure. The patient presented with years of globus sensation that had not been previously worked up. Associated symptoms included intermittent dysphagia and a chronic cough. The patient denied any throat pain, bleeding, hoarseness of voice, weight loss, and neck fullness. The patient had a significant 60-pack-year smoking history. 

**Figure 1:**
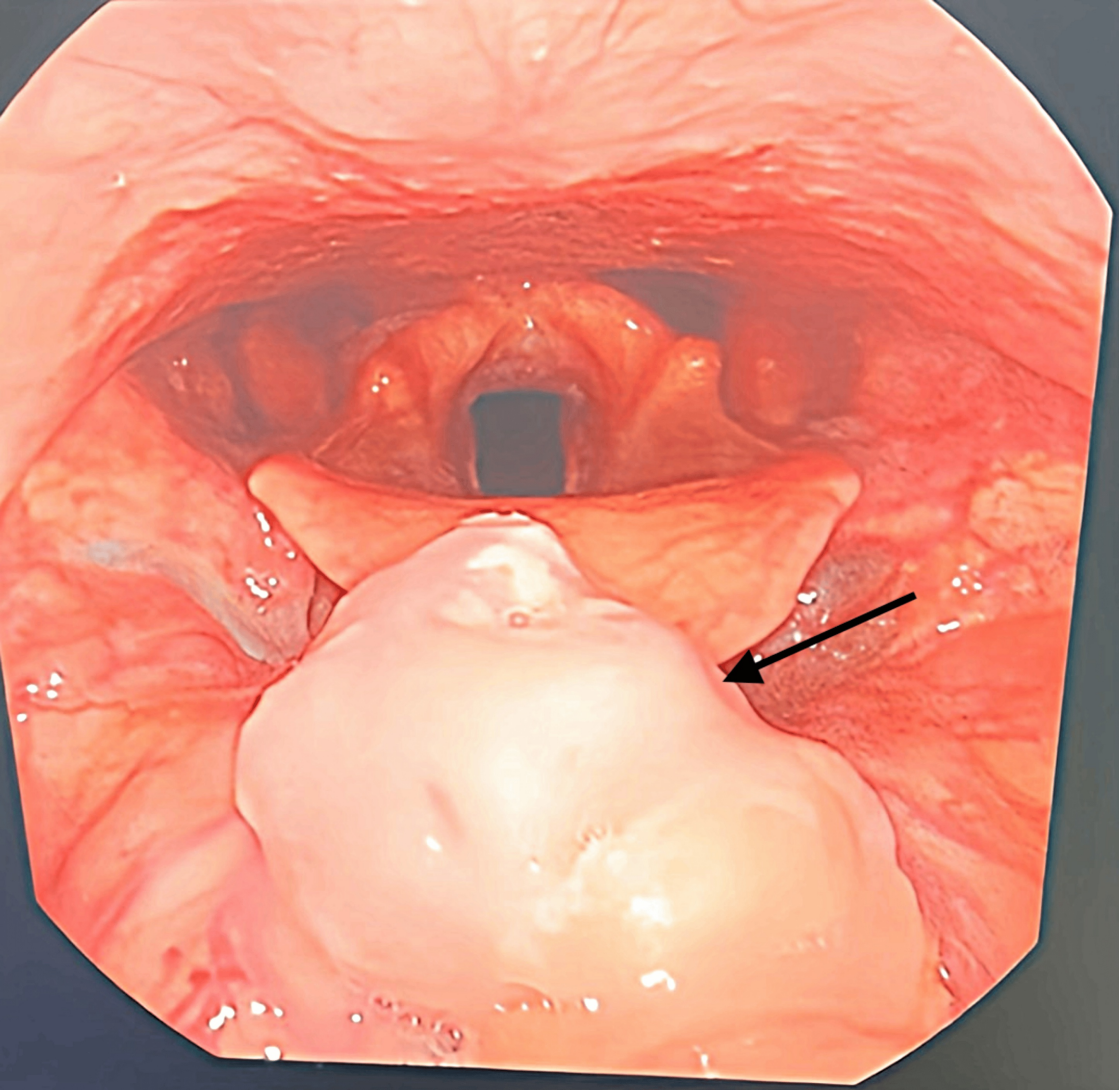
Oropharynx image of the patient Pedunculated midline mass (3-4 cm) at the root of the base of the tongue (BOT) indicated by the arrow.

On clinical examination, the lips and oral cavity were moist without any lesions and displayed appropriate mobility. The oropharynx showed pink smooth mucosa with smooth posterior pharyngeal walls. The hard and soft palate was intact without the presence of bony prominences, and the uvula was midline. On palpation of the neck, there was no evidence of a mass or lymphadenopathy. The thyroid gland was palpated with an anterior approach and did not reveal any nodules, enlargement, or masses. Based on the patient's history and prior bronchoscopy results, a flexible fiberoptic endoscope was performed to visualize the nasopharynx, oropharynx, hypopharynx, and larynx. In the oropharynx, there was a 3-4 cm pedunculated midline mass at the root of the base of the tongue (BOT) that was positioned posterior to the foramen cecum (Figure 1). After visual inspection of the mass with laryngoscopy, a biopsy was taken and sent for routine live pathology.

**Figure 2:**
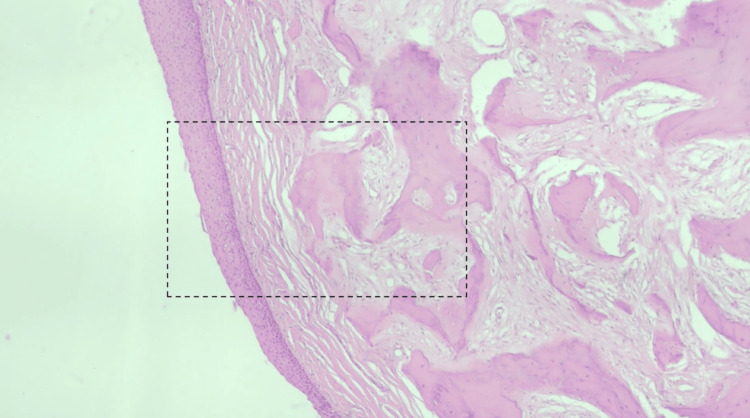
Low power magnification of biopsy specimen Hematoxylin and eosin (H&E) stain was used for this specimen. The dotted rectangular line indicates the section that was used for the high power magnification.

**Figure 3:**
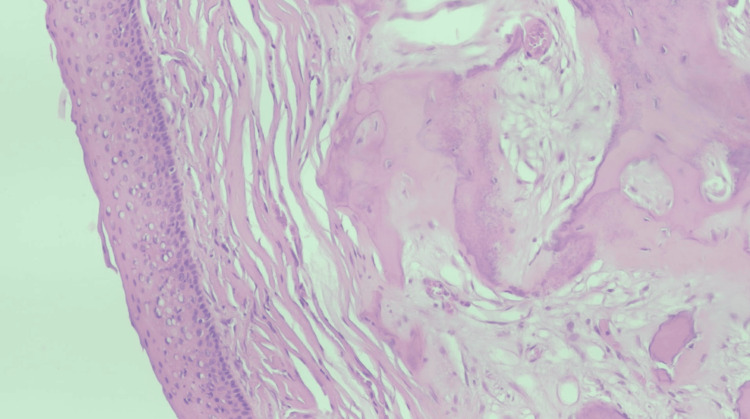
High power magnification of the biopsy specimen Hematoxylin and eosin (H&E) stain was used for this specimen.

Due to the unanticipated nature of the mass, and after decalcification, the biopsy specimen consisted of four firm, smooth-surfaced, gray-tan pieces with an aggregate measurement of 1.8 x 1.8 x 0.8 cm. The pathology report shared that benign-appearing squamous epithelium was present with associated fragments of spiculated bone (Figures 2, 3). There was no apparent evidence of high-grade atypia or malignancy. This description is consistent with a choristoma. Furthermore, the bony component exhibited a reparative type appearance with intermittent areas of a mixed inflammatory response that was secondarily present. The pathology diagnosis was confirmed by a second pathologist who agreed with the diagnosis of a choristoma. 

As a result of the biopsy, globus sensation, and the patient's request to have the mass removed, a planned surgical debulking of the mass using direct laryngoscopy and a combination of cup-biting forceps and microlaryngeal scissors. The goal of this procedure was to excise and debulk the lesion down to the base of the tongue, leave a subcentimeter stalk to track recurrence, and not disrupt the normal architecture of the tongue. It was also planned to leave a portion of the stalk so that the mass could be found at a later date for transoral robotic excision if the confirming intraoperative biopsy results differed from the original biopsy. Once the laryngoscope was placed into the patient's mouth, the base of the tongue was brought into view. A 3-4 cm pedunculated mass was visualized which was growing in the central portion of the base of the tongue posterior to foramen cecum and vallate papillae. The mass was not ulcerated but was firm, fibrotic, and very pedunculated. After the mass was debulked, an intraoperative confirmatory biopsy was taken, which later confirmed the diagnosis. This patient was seen one week, one month, and three months postoperatively. During these office visits, a flexible fiberoptic endoscope was used to visualize the surgical site, which showed no recurrence of the lesion. Additionally, the patient no longer had any complaints of globus sensation. Follow-up will continue to occur at one-year intervals as long as this patient remains asymptomatic without any evidence of recurrence.

## Discussion

According to Veni et al., a choristoma can be described formally as an ectopic anomaly in which histologically normal tissue is found in an aberrant anatomical site not naturally associated with that type of tissue [10]. Krolls et al. first coined the term "osseous choristoma" when they encountered intraoral lesions with bony fragments and observed different features compared to osteomas [5]. Intraoral osseous choristomas are exceedingly rare, with less than 100 reported cases since 2020, according to the most recent systematic review written by Shareef et al. [11]. Also, 70% of the reported cases occurred in females with an average age of 28.1 years [11,12]. As mentioned, most cases occur in the posterior third of the tongue and are positioned in front of the foramen cecum [8]. When analyzing these data points collectively, it becomes evident that the rarity of this present case is primarily attributable to the age and gender presentation of the patient, as well as the location in which the mass was found.

The pathogenesis of choristomas remains unknown and is a topic of opinion rather than a debate among medical experts. Several theories have been proposed to explain the development of these osseous lesions. Previously, researchers theorized that these lesions occur due to a congenital malformation derived from the brachial arches I, II, and III, trapped in the facial region [13,14]. Other alternative theories suggest that local inflammation, chronic irritation, or trauma sets off a reactive ossification process that leads to the formation of a mass [15,16]. These two proposed theories remain the most favored pathogenesis found within the literature. However, neither of these theories has definitively been proven, nor do they explain the predilection for females and why choristomas appear elsewhere in the head and neck region.

Surgical excision continues to be the standard treatment for these rare intraoral lesions. For BOT lesions, advances in oropharyngeal surgery have provided a plethora of surgical approaches that allow access to these enclosed masses. These surgical approaches to BOT lesions include transorally, suprahyoid pharyngotomy, Lip-split mandibulotomy, and Pull-through technique with visor incision [17]. The selection of the surgical approach is patient-centered and can vary case by case. Regardless of how the osseous choristoma is removed, recurrence is extremely unlikely, as there have only been three reported recurrences that have been reported [18].

## Conclusions

This case report outlines the diagnosis and management of a rare osseous choristoma in a 63-year-old male, an age and demographic that diverges from the typical patient profile for this condition. Intraoral osseous choristomas are exceedingly rare entities, often localized to the posterior third of the tongue and predominantly affecting younger females, thus making the occurrence in our elderly male patient particularly unusual. Furthermore, the lesion's placement behind the foramen cecum rather than in front of it adds an additional layer of rarity. The differential diagnosis for this case includes lingual tonsil hypertrophy, oropharyngeal squamous cell carcinoma, and papilloma. In conclusion, this case adds a valuable example to the limited literature on osseous choristomas, particularly those occurring in atypical patient demographics and locations. It also emphasizes the need for continued exploration into the pathogenesis of ectopic osseous formations and underlines the importance of tailored surgical strategies to manage such rare presentations effectively.

## References

[1] Chang H, Ahn Y, Lim YS, Hah JH (2009). Gastric choristoma of the oropharynx. Clin Exp Otorhinolaryngol.

[2] Rafizadeh SM, Ghadimi H, Nozarian Z (2020). Phakomatous choristoma of the eyelid and orbit: a case report. Pediatr Dev Pathol.

[3] Varshini K A, Desai A, Sahana BA, Kumar N (2023). A rare case of glial choristoma arising from the vomer in association with cleft palate. Cleft Palate Craniofac J.

[4] Rinaldo A, Ferlito A, Devaney KO (2004). Salivary gland choristoma of the middle ear. A review. ORL J Otorhinolaryngol Relat Spec.

[5] Krolls SO, Jacoway JR, Alexander WN (1971). Osseous choristomas (osteomas) of intraoral soft tissues. Oral Surg Oral Med Oral Pathol.

[6] Arens P, Ullrich A, Olze H, Uecker FC (2019). Extraoral osseous choristoma in the head and neck region: case report and literature review. Case Rep Otolaryngol.

[7] West CB Jr, Atkins JS Jr (1988). Choristomas of the intraoral soft tissues. Otolaryngol Head Neck Surg.

[8] Gorini E, Mullace M, Migliorini L, Mevio E (2014). Osseous choristoma of the tongue: a review of etiopathogenesis. Case Rep Otolaryngol.

[9] Adhikari BR, Sato J, Morikawa T (2016). Osseous choristoma of the tongue: two case reports. J Med Case Rep.

[10] Veni AC, Ashokan K, Sekar KC, D P, Sundaram KS, Aniyan Y (2020). Osseous choriostoma of the upper lip. Iran J Otorhinolaryngol.

[11] Shareef ZJ, Shareef SJ, Kerndt CC, Aughenbaugh A, Di Ponio A (2020). Lingual osseous choristoma: a comprehensive systematic review of lesion presentation, histology, and morphology. Spartan Med Res J.

[12] Macêdo MB, Borges SM, Macêdo P, Borges PM (2018). Lingual osteoma as a fortuitous finding on a boy with post-adenoidectomy inflammatory pseudotumor. Oral Maxillofac Surg Cases.

[13] Engel P, Cherrick HM (1976). Extraosseous osteomas of the tongue. J Oral Med.

[14] Gkouveris I, Kapranos N, Mitrou GG (2023). Lingual osseous choristoma: case report and literature review of this rare entity. J Clin Exp Dent.

[15] Heinz MJ, Peters SM, Caruana SM, Yoon AJ (2017). Lingual osseous choristoma of the tongue base: unusual presentation of a rare entity. Case Rep Otolaryngol.

[16] Benamer MH, Elmangoush AM (2007). Lingual osseous choristoma case report and review of literature. Libyan J Med.

[17] Van Abel KM, Moore EJ (2013). Surgical management of the base of tongue. Oper Tech Otolayngol Head Neck Surg.

[18] Gregoire CE, Davis CM, Bullock MJ (2015). Recurrent Osseous Choristoma Involving the Mandibular Buccal Vestibule: A Case Report.

